# Effect of indocyanine green near-infrared light imaging technique guided lymph node dissection on short-term clinical efficacy of minimally invasive radical gastric cancer surgery: a meta-analysis

**DOI:** 10.3389/fonc.2023.1257585

**Published:** 2023-09-11

**Authors:** Sen Niu, Yuan Liu, Da Li, Yufan Sheng, Ye Zhang, Zengyao Li, Songyun Zhao, Tong Wang

**Affiliations:** ^1^ Department of General Surgery, Wuxi People’s Hospital Affiliated To Nanjing Medical University, Wuxi, China; ^2^ Department of Neurosurgery, Wuxi People’s Hospital Affiliated To Nanjing Medical University, Wuxi, China

**Keywords:** indocyanine green, gastric cancer resection, lymph node dissection, laparoscopy, robotics

## Abstract

**Objective:**

In recent years, the utilization of indocyanine green near-infrared (ICG NIR) light imaging-guided lymph node dissection in the context of minimally invasive radical gastric cancer has emerged as a novel avenue for investigation. The objective of this study was to assess the influence of employing this technique for guiding lymph node dissection on the short-term clinical outcomes of minimally invasive radical gastric cancer surgery.

**Methods:**

The present study conducted a comprehensive search for short-term clinical outcomes, comparing the group undergoing ICG NIR light imaging-guided lymph node dissection with the control group, by thoroughly examining relevant literature from the inception to July 2023 in renowned databases such as PubMed, Embase, Web of Science, and Cochrane Library. The primary endpoints encompassed postoperative complications, including abdominal infection, abdominal bleeding, pneumonia, anastomotic fistula, and overall incidence of complications (defined as any morbidity categorized as Clavien-Dindo class I or higher within 30 days post-surgery or during hospitalization). Additionally, secondary outcome measures consisted of the time interval until the initiation of postoperative gas and food intake, as well as various other parameters, namely postoperative hospital stay, operative time, intraoperative blood loss, total number of harvested lymph nodes, and the number of harvested metastatic lymph nodes. To ensure methodological rigor, the Cochrane Collaboration Risk of Bias Tool and the Newcastle-Ottawa Scale (NOS) were employed to assess the quality of the included studies, while statistical analyses were performed using Review Manager 5.4 software and Stata, version 12.0 software.

**Results:**

A total of 19 studies including 3103 patients were ultimately included (n=1276 in the ICG group and n=1827 in the non-ICG group). In this meta-analysis, the application of ICG near-infrared light imaging in minimally invasive radical gastric cancer surgery effectively improved the occurrence of postoperative Clavien-Dindo grade II or higher complications in patients (RR=0.72, 95% CI 0.52 to 1.00) with a statistically significant P=0.05; in reducing intraoperative blood loss and shortening While reducing intraoperative blood loss and shortening postoperative hospital stay, it could ensure the thoroughness of lymph node dissection in minimally invasive radical gastric cancer surgery (MD=5.575, 95% CI 3.677-7.473) with significant effect size (Z=5.76, p<0.00001).

**Conclusion:**

The utilization of indocyanine green near-infrared light imaging technology in the context of minimally invasive radical gastric cancer surgery demonstrates notable efficacy in mitigating the occurrence of postoperative complications surpassing Clavien-Dindo grade II, while concurrently augmenting both the overall quantity of lymph node dissections and the identification of positive lymph nodes, all the while ensuring the preservation of surgical safety. Furthermore, the implementation of this technique proves particularly advantageous in the realm of robotic-assisted radical gastric cancer surgery, thus bearing significance for enhancing the short-term prognostic outcomes of patients.

## 1 Introduction

As a highly heterogeneous solid tumor, gastric cancer is the third most common cause of cancer-related deaths worldwide, and its occurrence and development are related to numerous factors such as genetics and environment (1, 2). Since its introduction by Kitano et al. in 1994, laparoscopic radical surgery for distal gastric cancer in Japan marked a pivotal milestone, leading to the widespread adoption of minimally invasive radical surgery for gastric cancer in clinical practice. Over the course of more than two decades of development (3, 4), this approach has evolved significantly. In recent years, propelled by advancements in laparoscopic and surgical robotic instruments as well as technological breakthroughs, minimally invasive gastric cancer surgery has progressively embraced the realm of precision medicine. Consequently, precise and facile tumor localization and lymph node navigation within the context of minimally invasive surgical procedures, alongside systematic and comprehensive lymph node dissection and preservation of secure anastomotic blood flow, emerge as pivotal factors crucial to both the immediate and long-term prognoses of patients (5).

Indocyanine green (ICG) serves as a biocompatible near-infrared (NIR) photocontrast agent, responsive to external light within the wavelength range of 750-800 nm, emitting NIR light at approximately 840 nm. Its tissue penetration depth spans between 0.5 and 1.0 cm (6). Following local administration *via* submucosal or plasma membrane injection, ICG undergoes distinct metabolic pathways. A portion binds to tissue albumin and remains within the local tissues, facilitating prompt tumor localization and identification of diverse tissue types through observation of fluorescence levels. Another portion is absorbed by the lymphatic system, subsequently binding to lymphatic albumin. This fraction is then transported to the lymph nodes and ultimately reenters the bloodstream (6). Moreover, intraoperative intravenous administration of ICG proves advantageous in evaluating the blood supply to various structures such as the gastric wall, intestinal wall, anastomotic site, spleen, and liver. This application aids in reducing the incidence of anastomotic leakage (7, 8). ICG NIR light imaging, as a novel surgical navigation technique, has yielded encouraging outcomes in facilitating the localization of anterior lymph node clearance in various malignancies, including non-small cell lung cancer, breast cancer, and other tumor types (9, 10). Through the proficient utilization of ICG fluorescence (ICG FL) imaging within laparoscopic devices, an increasing number of surgeons have discovered that ICG NIR imaging exhibits superior tissue penetration capabilities, enabling enhanced identification of lymph nodes within hypertrophic adipose tissue compared to other dyes utilized under visible light. Consequently, ICG NIR imaging presents a promising avenue for exploration and application within the realm of lymph node dissection for minimally invasive radical gastric cancer, garnering substantial attention both domestically and internationally (11–13). Nevertheless, in the current clinical landscape, the utilization of ICG NIR imaging technology as a guiding tool for lymph node dissection in minimally invasive radical gastric cancer remains in the exploratory phase, lacking a standardized approach. Additionally, there exists a learning curve associated with proficiently implementing this technology, and the requirement of an expensive NIR imaging system poses a challenge, limiting its widespread adoption in many medical centers. Consequently, the clinical efficacy of this technology in the context of patients undergoing minimally invasive radical gastric cancer surgery remains uncertain. The objective of this study was to conduct a meta-analysis examining the short-term clinical outcomes of employing this technique for guided lymph node dissection in minimally invasive radical gastric cancer, with the aim of assessing both its advantages and limitations.

## 2 Information and methods

### 2.1 Study registration

The protocol for this systematic review was registered with PROSPERO under the registration number CRD42023429689.

### 2.2 Search strategy databases

PubMed, Embase, Web of Science, Cochrane Library databases. Search terms: indocyanine green, ICG, stomach neoplasms, gastric cancer, gastric carcinoma, stomach cancer, lymphadenectomy, lymph node dissection, etc (**Table S1**). Language of literature: English. Search time: start to July 2023.

### 2.3 Exclusion and inclusion criteria

#### 2.3.1 Inclusion criteria

①Study type: cohort study and randomized controlled trial; ②Study population: patients who underwent laparoscopic or robotic minimally invasive radical gastric cancer surgery and whose postoperative pathology was clearly diagnosed as gastric cancer; ③Outcome indicators: the main outcome indicators were comparing short-term postoperative clinical outcomes in the ICG and non-ICG groups including postoperative abdominal infection, abdominal bleeding, pneumonia, anastomotic fistula, total complication rate (any morbidity classified as Clavien-Dindo grade I or higher occurring within 30 days of surgery or during hospitalization), incidence of Clavien-Dindo grade II or higher complications, time to first postoperative venting and feeding, and the remaining outcome indicators including postoperative hospital stay, time to operative time, intraoperative blood loss, and total number of harvested lymph nodes.

#### 2.3.2 Exclusion criteria

①type of literature: review, non-comparative study, conference report, case report, and other types of literature that do not match; ②study subjects: combined with other malignancies or unable to tolerate surgery; ③outcome indicators: indocyanine green infrared light imaging technique to guide lymph node dissection is not described or the outcome indicators do not match; ④quality of literature: poor experimental design, lack of necessary computational data, or low quality of literature.

### 2.4 Literature screening, quality assessment and data extraction

Screening of literature, quality assessment and data extraction were done independently by two researchers. In case of disputes, two researchers had to discuss and agree with a third researcher. Researchers completed the literature screening process using EndNote software, reading abstracts and full text when necessary.The Cochrane Collaboration Risk of Bias Tool and the Newcastle-Ottawa Scale (NOS) were used to assess the risk of bias and quality assessment of included randomized controlled trials (RCTs) and observational studies, respectively. Experimental data were extracted by reading the full text of the literature. Extracted data included study characteristics (authors, year of publication, study country, study interval, study design and sample size), clinical characteristics (age, gender, body mass index, surgical method, history of neoadjuvant radiotherapy, American Society of Anesthesiologists score, pathological tumor variables), different methods of ICG use and outcome measures. The primary outcome indicators were comparison of short-term postoperative clinical outcomes including postoperative abdominal infection, abdominal bleeding, pneumonia, anastomotic fistula, overall complication rate (morbidity of any classification of Clavien-Dindo grade I or higher occurring within 30 days after surgery or during hospitalization), Clavien-Dindo grade II or higher complication rate, and time to first postoperative venting and feeding, and the remaining outcome indicators included postoperative length of stay, operative time, intraoperative blood loss, and total number of harvested lymph nodes. The original dataset utilized in the study can be found in **Table S2**.

### 2.5 Statistical analyses

All statistical analyses were performed using Review Manager 5.4 software (The Cochrane Collaboration, The Nordic Cochrane Centre, Copenhagen, Denmark) and Stata, version 12.0 software (StataCorp LP. College Station, TX, USA) were performed. Effect sizes for dichotomous and continuous data were expressed as relative risk (RR) and mean difference (MD), respectively, and 95% confidence intervals (95% CI) were calculated for both, respectively. The magnitude of heterogeneity between studies was tested using the χ² test and I² quantification as well as forest plots. In all analyses, p < 0.05 was considered significant. Heterogeneity was ignored when I² < 50%, moderate heterogeneity when I² = 50% or > 50% ~ 70%, and significant heterogeneity when I² > 70%. If there was significant heterogeneity among the findings (p < 0.05 and I² ≥ 50%) and the cause of heterogeneity could not be explored by subgroup, sensitivity analysis, or Meta regression, a random-effects model was selected to combine effect sizes; otherwise, fixed-effects combined effect sizes were performed. Final tests for publication bias were performed using funnel plot, Egger’s method and Trim’s method.

## 3 Results

### 3.1 Literature search results

**Figure 1:**
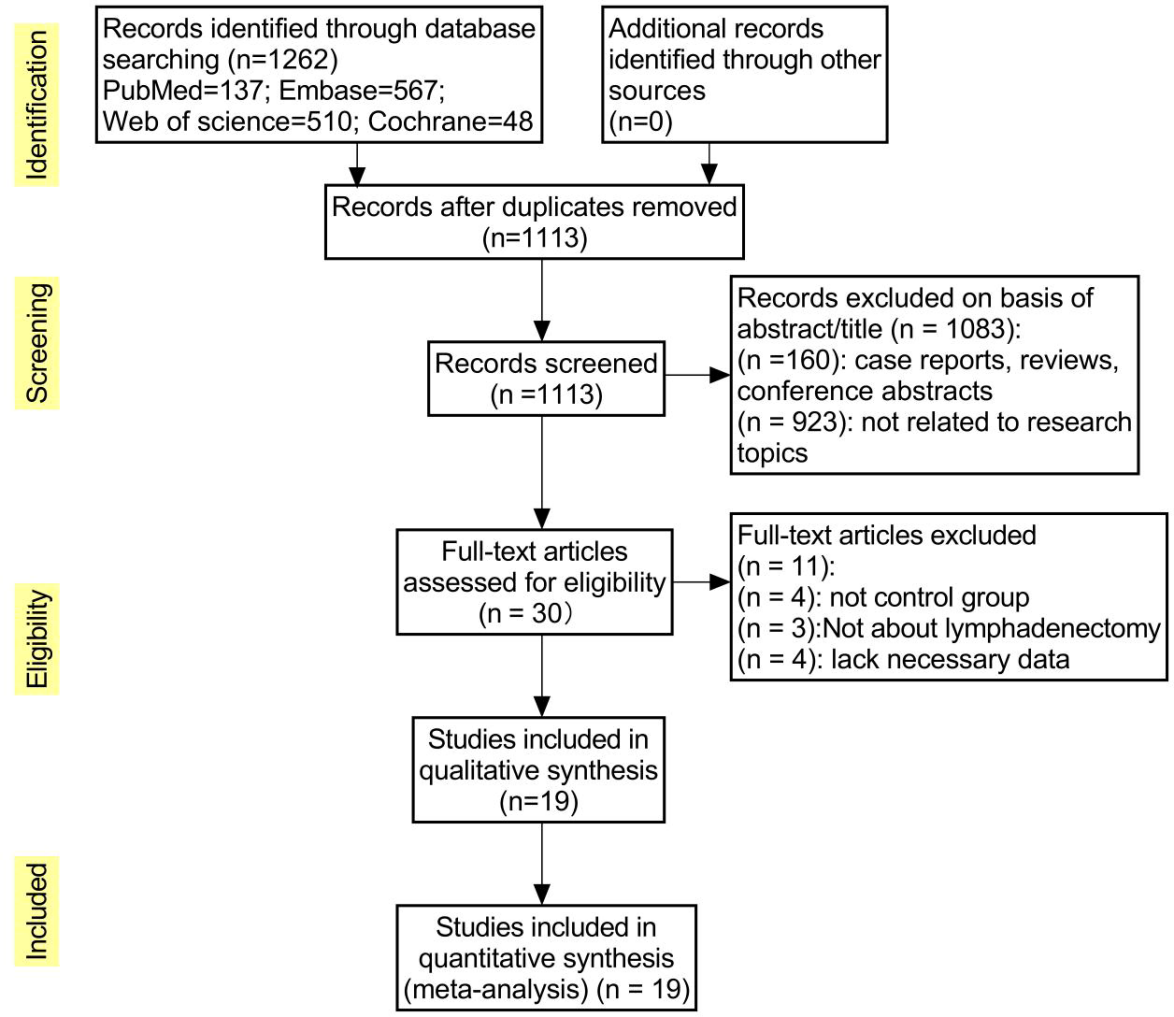
Literature filtering process.

The initial database search yielded 1262 publications, and after excluding 149 duplicates, the remaining studies (n=1113) were screened for title and abstract relevance; 1083 were excluded because they were case reports, reviews, or conference abstracts (n=160) or not related to the study topic (n=923). The remaining 30 full-text literature articles were searched and evaluated, and were excluded because some studies did not have a control group (n=4), ICG was not applied to lymph node dissection (n=3), or lacked necessary data (n=4), resulting in the inclusion of 19 studies, including 2 randomized controlled trials, 2 prospective cohort studies, and 15 retrospective cohort studies. The flow chart of literature screening is shown in **Figure 1**.

### 3.2 Baseline characteristics and quality evaluation of the literature

Nineteen studies were included from five different countries, including Korea, China, Spain, Italy, and Japan, with a total sample size of 3103 patients. 14 studies reported laparoscopic-assisted radical gastric cancer surgery, 3 studies reported robotic-assisted radical gastric cancer surgery, and 2 studies mixed laparoscopic and robotic-assisted radical gastric cancer surgery. All studies divided the sample into ICG-guided lymph node dissection group and control group. Baseline characteristics of the included studies are shown in **Table 1**. The Cochrane Collaboration Risk of Bias Tool (33) and the Newcastle-Ottawa scale (34, 35) were used to assess the quality of the literature of the included randomized controlled trials (RCTs) and observational studies, respectively, and the results are shown in **Tables 2**, **3**.

**Table 1:** Baseline characteristics of the included literature.

	Author	Area	Year	Type of group	Study interval	Number	Age (years)	Sex	Body mass index (kg/m2)	gastrectomy	Adjuvant chemotherapy	ASA >2	gastrectomy extent	tumor size(mm)	Clinical stage included	Extent of lymphadenec tomy	ICG injection	Imaging system	Type of study
NO1	Alrashidi, N (14).	Korea	2022	ICG	April 2013 - December 2020	32	62.5 ± 14.7	Female11Male21	21.9(6.3)	Lap12Robot20	0	13	TG32	35.7 ± 19.5	cT1-cT4a, N0/+, M0		SMA	A Pinpoint^®^ fluorescence imaging system (Novadaq, Mississauga, Ontario, Canada)	NRCT
				non-ICG		36	59.9 ± 14.1	Female28Male8	21.4(3.9)	Lap28Robot8	0	13	TG36	31.2 ± 18.9	cT1-cT4a, N0/+, M0				NRCT
NO2	Chen, Q. Y (15).	China	2020	ICG	November 2018 -July 2019.	129	57.8 ± 10.7	Female43Male86	23.2(3.2)	Lap129Robot0	0	0	DG71TG58		cT1-cT4a, N0/+, M0	D2	SMA	A NOVADAQ fluorescence surgical system (Stryker Co., Kalamazoo, MI, USA)	RCT
				non-ICG		129	60.1 ± 9.1	Female42Male87	22.8(3.1)	Lap129Robot0	0	0	DG43TG86		cT1-cT4a, N0/+, M0	D2		green fluorescence mode of fluorescence laparoscopy	RCT
NO3	Chen, X (16).	China	2022	ICG	March 2019-December 2020	18	59.11 ± 5.72	Female6Male12	23.68 ± 3.19	Lap189Robot0		2	DG12TG6		cT1-cT4a, N0/+, M0		SMA		NRCT
				non-ICG		38	60.42 ± 8.09	Female10Male28	22.72 ± 3.08	Lap38Robot0		5	DG23TG15		cT1-cT4a, N0/+, M0				NRCT
NO4	Cianchi, F (17).	Italy	2020	ICG	June 2014 - June 2018	37	72.2 ± 9.8	Female15Male22	23.3 ± 3.07	Lap0Robot37	2	12	TG7STG30	38 ± 19	cT1-cT3, N0/+, M0	D2	SMA	A near-infrared camera system (Firefly Fluorescence Imaging Scope; Intuitive Surgical, Sunnyvale, CA)	NRCT
				non-ICG		37	72.4 ± 8.9	Female16Male21	23.2 ± 3.04	Lap0Robot37	2	12	TG12STG25	39 ± 21.9	cT1-cT3, N0/+, M0	D2			NRCT
NO5	Huang, Z. N (18).	China	2021	ICG	February 2010 - October 2020	94	60.04 ± 10.51	Female25Male69		Lap94Robot0	94		DG15TG79	43.6 ± 18.4	cT1-4, N0-3, M0	D2	SSA	The NOVADAQ fluorescence surgical system (Stryker Corp., Kalamazoo, MI, USA)	NRCT
				non-ICG		94	60.47 ± 9.92	Female20Male74		Lap94Robot0	94		DG12TG82	46.3 ± 22.8	cT1-4, N0-3, M0	D2			NRCT
NO6	Kwon, I. G (19).	Korea	2019	ICG	August 30, 2013- July 21, 2014	40	52.2 ± 11.7	Female19Male21	23.3 ± 2.6	Lap0Robot40	0		DG34TG6	25.4 ± 17.7	cT1-2,N0-1,M0	D1+,D2	SMA	The da Vinci Si Surgical System (Intuitive) equipped with the Firefly mode	NRCT
				non-ICG		40	52.1 ± 11.3	Female21Male19	23.3 ± 3.4	Lap0Robot40	0		DG32TG8	21.8 ± 13.3	cT1-2,N0-1,M0	D1+,D2			NRCT
NO7	Lan, Y (20).	China	2017	ICG	January 2011 - March 2016	14	66.0 ± 12.4	Female7Male7	24.0 ± 4.1	Lap0Robot14			STG11TG3	37± 17	cT1-cT4a, N0/+, M0	D2	SSA(n=9)SMA(n=5)	A near-infrared (NIR) imaging system which was equipped in the da Vinci Si Surgical system.	NRCT
				non-ICG		65	67.8 ± 15.6	Female27Male38	24.4 ± 3.1	Lap0Robot65			STG59TG6	3.4 ± 1.6	cT1-cT4a, N0/+, M0	D2,<D2			NRCT
NO8	Lee, S (21).	Korea	2022	ICG	January 2013- December 2018	74	56.1 ± 13	Female29Male45	23.5 ± 4.2	Lap24Robot50	0	15	TG74	Median34	cT1-cT4a, N0/+, M0	D2 + No. 10 lymph node	SMA	A Pinpoint^®^ fluorescence imaging system (Novadaq, Mississauga, Ontario, Canada)	NRCT
				non-ICG		94	56.3 ± 13.5	Female39Male55	22.9 ± 2.8	Lap75Robot19	0	19	TG94	Median40	cT1-cT4a, N0/+, M0	D2 + No. 10 lymph node			NRCT
NO9	Liu, M (22).	China	2020	ICG	August 2017 - November 2019	61	55.11 ± 10.76	Female28Male33	23.75 ± 3.49	Lap61Robot0	0	0	DG61	Median26	cT1-cT4a, N0/+, M0	D2	SMA	The NOVADAQ fluorescence surgical system (Stryker Corp., Kalamazoo, MI, USA)	NRCT
				non-ICG		75	58.40 ± 10.71	Female28Male47	23.51 ± 2.51	Lap75Robot0	0	0	DG75	Median26.8	cT1-cT4a, N0/+, M0	D2			NRCT
NO10	Lu, X (23).	China	2021	ICG	July 2015 - October 2019	28	57.96 ± 12.66	Female9Male19	22.25 ± 2.32	Lap28Robot0		15	PG3DG10TG15		cT1-cT3, N0/+, M0	D2	SMA	The Endoscopic Fluorescence Imaging System (PINPOINT, NOVADAQ, Mississauga, ON, Canada)	NRCT
				non-ICG		28	59.17 ± 9.17	Female8Male20	22.86 ± 2.73	Lap28Robot0		15	PG6DG13TG9		cT1-cT4a, N0/+, M0	D2			NRCT
NO11	Ma, S (24).	China	2019	ICG	December 2018-August 2019	38	59.7 ± 9.1	Female9Male29	23.7 ± 2.9	Lap38Robot0		4	PG4DG30TG4		cT2-cT4, N0/+, M0	D1+,D2	SMA	The Storz Fluorescent Laparoscopic Equipment	NRCT
				non-ICG		44	57.3 ± 12.0	Female13Male31	23.1 ± 3.1	Lap44Robot0		6	PG7DG31TG6		cT2-cT4, N0/+, M0	D1+,D2			NRCT
NO12	Maruri, I (25).	Spain	2022	ICG	July 2019-January 2022	17	Median63.6	Female10Male7	Median25.0	Lap17Robot0	3	8	STG7TG10		cT1-cT4a, N0/+, M0	D1.5,D2	SMA		NRCT
				non-ICG		17	Median66.4	Female2Male15	Median26.0	Lap17Robot0	10	9	STG5TG12		cT1-cT4a, N0/+, M0	D1.5,D2			NRCT
NO13	Park, S (26).	Korea	2020	ICG	July 2017 - June 2018	20	60.10 ± 11.09	Female5Male15	24.89 ± 3.22	Lap20Robot0			DG20	30.3 ± 12.5	cT1-cT4a, N0/+, M0	D1+,D2	SMA	A real-time endoscopic NIR imaging system (PINPOINT;Novadaq Inc., Mississauga, ON, Canada)	NRCT
				non-ICG		60	61.67 ± 11.47	Female15Male45	24.09 ± 2.80	Lap60Robot0			DG60	28.2 ± 16.0	cT1-cT4a, N0/+, M0	D1+,D2			NRCT
NO14	Puccetti, F (27).	Italy	2022	ICG	April 2015 - August 2021	38	Median69	Female16Male22	2 5 ± 7	Lap38Robot0	22		TG38		cT1-cT3, N0/+, M0	D2	SMA		NRCT
				non-ICG		64	Median70	Female24Male40	2 2 ± 9	Lap64Robot0	23		TG64		cT1-cT3, N0/+, M0	D2			NRCT
NO15	Tu, R (28).	China	2019	ICG	April 2017 - December 2017	39	58 ± 13	Female13Male26	23 ± 4	Lap39Robot0	0		PG2DG16TG21	40 ± 23	cT1-cT4a, N0/+, M0	D2	SMA		NRCT
				non-ICG		663	61 ± 11	Female172Male491	24 ± 4	Lap663Robot0	0		PG1DG299TG363	43 ± 33	cT1-cT4a, N0/+, M0	D2			NRCT
NO16	Ushimaru, Y (29).	Japan	2019	ICG	July 2015 - August 2017	84	66.2 ± 1.2	Female37Male47	22.92 ± 0.35	Lap84Robot0	0		DG84	42.6 ± 2.3	cT1-cT4a, N0/+, M0	D1+,D2	SMA	A NIR/ICG telescope and camera head system (IMAGE1 S™ System,KARL STORZ, Tuttlingen, Germany)	NRCT
				non-ICG		84	66.6 ± 1.2	Female38Male46	22.83 ± 0.35	Lap84Robot0	0		DG80TG4	38.7 ± 2.3	cT1-cT4a, N0/+, M0	D1+,D2			NRCT
NO17	Wei, M (30).	China	2022	ICG	January 2018 - August 2019	107	59.27 ± 8.99	Female50Male57	24.60 ± 3.41	Lap107Robot0	0	8	DG59TG48	40.3 ± 24.8	cT1-cT4a, N0/+, M0	D2	SMA	A NOVADAQ fluorescence surgical system (Stryker Co., Kalamazoo, MI, USA)	NRCT
				non-ICG		88	61.53 ± 10.30	Female40Male48	24.95 ± 2.65	Lap88Robot0	0	10	DG41TG47	40.9 ± 24.6	cT1-cT4a, N0/+, M0	D2			NRCT
NO18	Yoon, B. W (31).	Korea	2022	ICG	January 2010 - November 2020	21		Female6Male15		Lap21Robot0			DG21	25.8 ± 15.8	cT1-cT4a, N0/+, M0	D2	SMA		NRCT
				non-ICG		42		Female13Male29		Lap42Robot0			DG42	25.6 ± 15.6	cT1-cT4a, N0/+, M0	D2			NRCT
NO19	Zhong, Q (32).	China	2021	ICG	November 2018 - October 2020/December 2019 - October 2020	385	58.5 ± 10.7	Female126Male259	22.7 ± 3.2	Lap385Robot0	0		DG207TG178	39 ± 24	cT1-cT4a, N0/+, M0	D1, D1+, D2	SMA(n=256),SSA(n=129)	A NOVADAQ fluo-rescence surgical system (Stryker Corp., Kalamazoo, MI, USA)	RCT
				non-ICG		129	60.1 ± 9.1	Female42Male87	22.8 ± 3.1	Lap129Robot0	0		DG43TG86	44 ± 22	cT1-cT4a, N0/+, M0	D1, D1+, D2			RCT

ICG,indocyanine green; BMI, body mass index;Lap,laparoscopic-assisted radical gastric cancer surgery;Robot,Robot-assisted radical gastric cancer surgery;ASA, American Society of Anesthesiologists; DG, distal gastrectomy, TG, total gastrectomy, PG, proximal gastrectomy, STG, subtotal gastrectomy; SMA, submucosal injection; SSA, subplasmic injection; TNM stage according to AJCC 8th.

**Table 2:** Evaluation of the quality of literature included in randomized controlled trials.

Study	Random sequence generation	Allocation concealment	Blinding of participants and personnel	Blinding of outcome assessment	Incomplete outcome data	Selective reporting	Other bias
Chen, Q. Y. 2020	Low risk	Low risk	High risk	High risk	Unclear risk	Low risk	Low risk
Zhong, Q. 2021	Low risk	Low risk	Unclear risk	Unclear risk	Low risk	Low risk	Unclear risk

**Table 3:** Newcastle-Ottawa Scale (NOS) scores for the included non-randomized controlled trials.

Study	Selection	Comparability	Outcome	Score
Alrashidi, N. 2022	★★★★	★★	★★	8
Chen, X. 2022	★★★	★★	★★	7
Cianchi, F. 2020	★★★	★★	★★★	8
Huang, Z. N. 2021	★★★★	★★	★★	8
Kwon, I. G. 2019	★★★	★★	★★★	8
Lan, Y. 2017	★★★	★	★★	7
Lee, S. 2022	★★★★	★★	★★	8
Liu, M. 2020	★★★	★★	★★★	8
Lu, X. 2021	★★★	★★	★★★	8
Ma, S. 2019	★★★★	★★	★★	8
Maruri, I. 2022	★★★	★★★	★	7
Park, S. 2020	★★★★	★★	★★	8
Puccetti, F. 2022	★★★	★★★	★	7
Tu, R. 2019	★★★	★★	★★	7
Ushimaru, Y. 2019	★★★	★★	★★★	8
Wei, M. 2022	★★★	★★	★★	8
Yoon, B. W. 2022	★★★★	★★	★	7

### 3.3 Clinical outcome assessment

#### 3.3.1 Short-term postoperative prognosis

The incidence of short-term postoperative complications in patients is a key indicator to assess the success of the procedure. The outcome effect measures covered in this Meta include mainly the total number of postoperative complications occurring, the incidence of Clavien-Dindo grade II or higher complications, in addition to specific comparisons of postoperative abdominal infection, abdominal bleeding, pneumonia, anastomotic fistula, postoperative gastric emptying disorder, and postoperative complications of intestinal obstruction.

**Figure 2:**
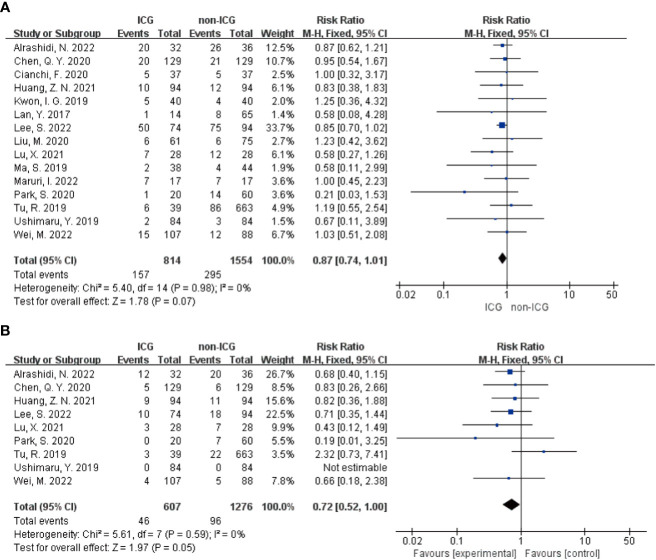
Forest plot assessment of short-term postoperative prognosis in the ICGFL group versus the non-ICGFL group. **(A)** total postoperative complication rate; **(B)** complication rate for Clavien-Dindo classification grade II or higher.

**Figure 3:**
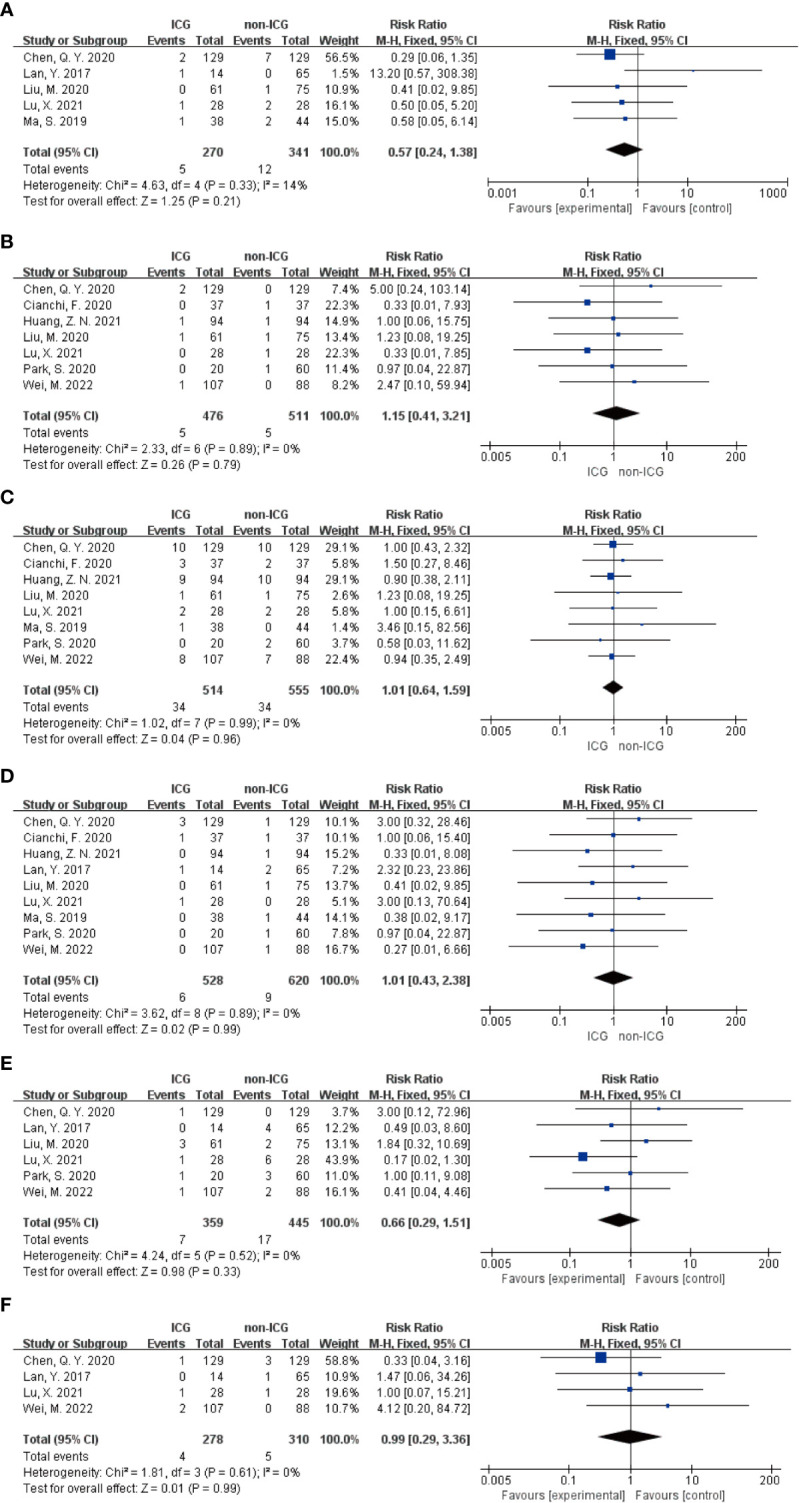
Forest plot assessment of short-term postoperative prognostic outcomes in the ICGFL and non-ICGFL groups. **(A-F)** in order of postoperative abdominal infection, abdominal bleeding, pneumonia, anastomotic fistula, postoperative gastric emptying disorder, and postoperative complications of concomitant intestinal obstruction.

Meta-analysis results regarding the total number of postoperative complications in the two groups showed that the 15 papers of this study, after heterogeneity test, I²=0% <50% and P=0.979 > 0.1 for Q-test, suggesting that there is no heterogeneity between the papers selected for this study (heterogeneity is not statistically significant), then the fixed effect was selected for the combined effect size; the fixed effect combined effect RR=0.866 (0.739 to 1.014), but not statistically significant Z=1.78, P=0.075 > 0.05, suggesting that the occurrence of total postoperative complications was not significantly improved in the ICG group compared with the non-ICG group (**Figure 2A**); therefore, this Meta pair covering Clavien-Dindo studies covering the incidence of complications above grade II were analyzed separately, and a total of 9 studies were included in the literature; the results of the forest plot showed that the heterogeneity test I²=0% < 50% and P=0.59 > 0.1 for the Q-test, suggesting that there was no heterogeneity between the literature selected for this study (heterogeneity was not statistically significant), and then fixed effects were selected for the combined effect size. In addition, nine studies used fixed effects for combined effects RR=0.72 (0.52 to 1.00) and were statistically significant Z=1.97, P=0.05, suggesting that for the incidence of Clavien-Dindo grade II or higher complications, the ICG group was significantly more effective than the non-ICG group for postoperative improved (**Figure 2B**). Meta-analysis results regarding postoperative abdominal infection, abdominal hemorrhage, pneumonia, anastomotic fistula, postoperative gastric emptying disorder, and postoperative complications of intestinal obstruction in patients in both groups, respectively, showed no heterogeneity among the studies included in the six data sets results, and fixed effects were selected for the combined effect sizes. However, there were no significant differences in the occurrence of postoperative abdominal infection, abdominal bleeding, pneumonia, anastomotic fistula, postoperative gastric emptying disorder, and postoperative complications of intestinal obstruction between the two groups of patients. The details are shown in the following figures (**Figures 3A–F**).

#### 3.3.2 Surgery and postoperative recovery

In this Meta-analysis regarding the assessment of surgery and postoperative recovery, the main components were the operative time, intraoperative blood loss, total number of lymph nodes cleared, total number of metastatic lymph nodes cleared, postoperative hospital stay, time to first postoperative gas, and time to first postoperative fluid intake.

**Figure 4:**
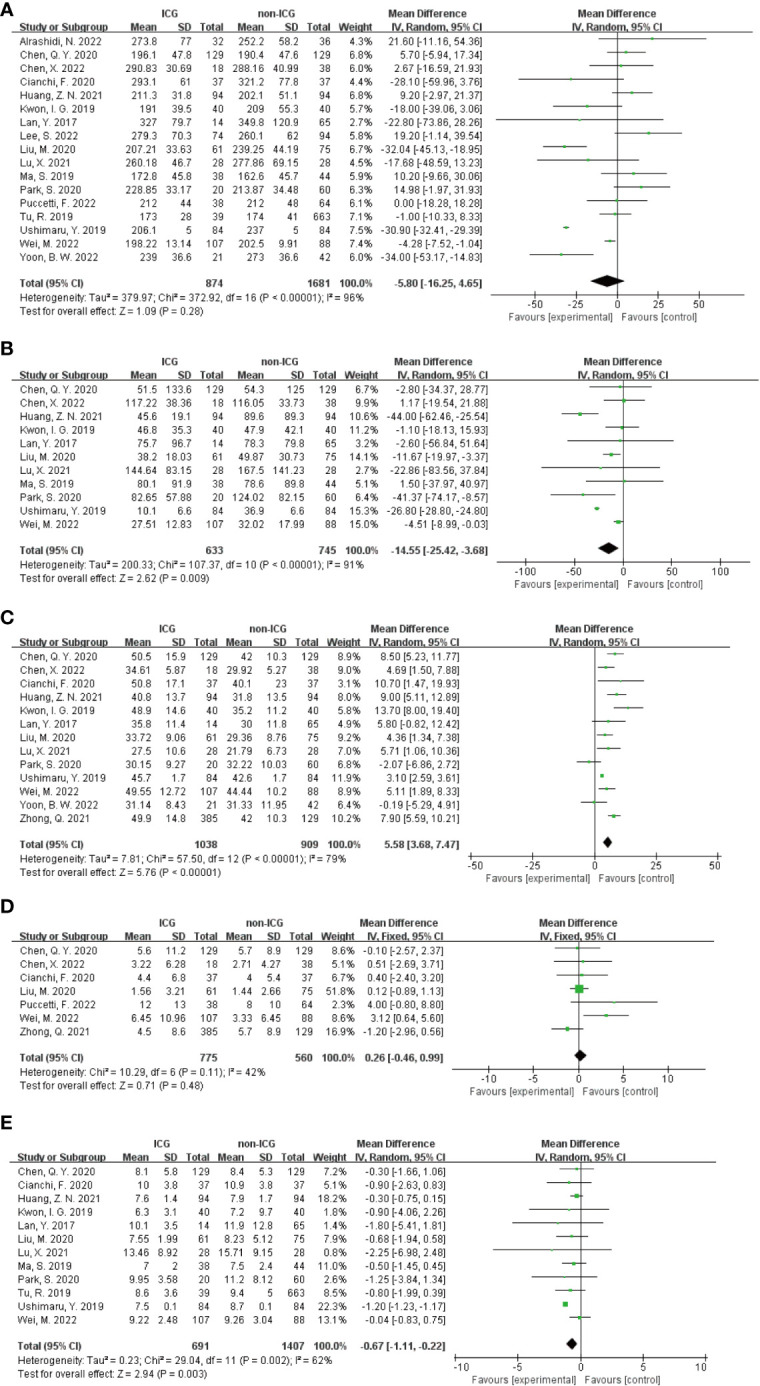
Forest plots to assess the surgical and postoperative recovery in the ICG and non-ICG groups. **(A–E)** are operation time, intraoperative blood loss, total number of lymph nodes dissected, total number of metastatic lymph nodes dissected, and postoperative hospital stay.

**Figure 5:**
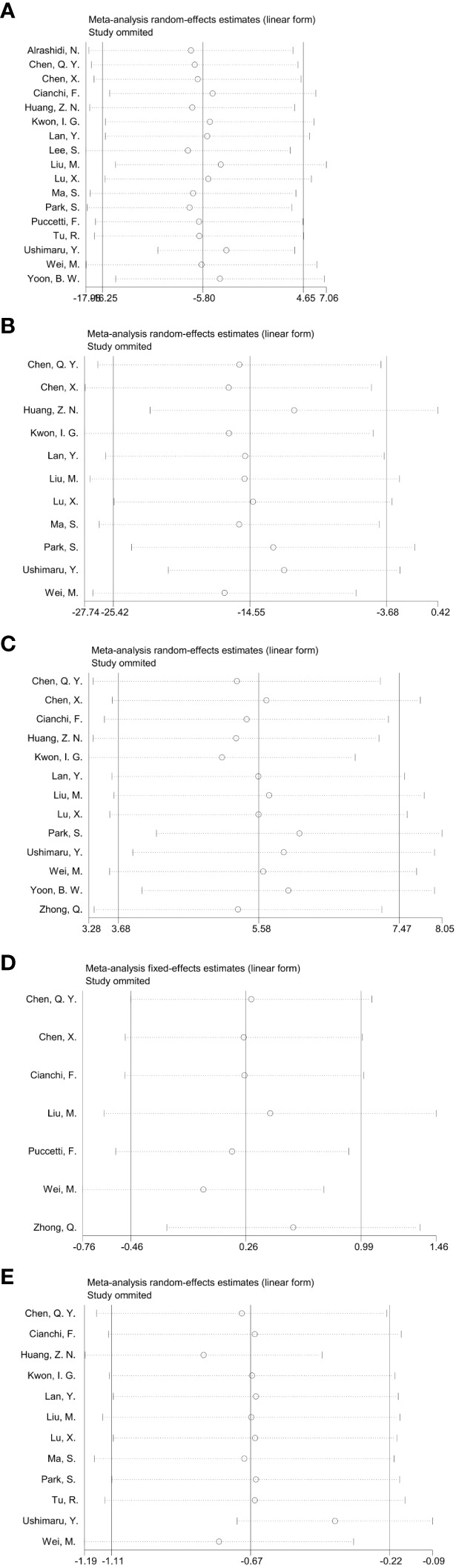
Sensitivity analysis of each group included in the study regarding surgery and postoperative recovery in the ICG group versus the non-ICG group. **(A–E)** in order of surgery time, intraoperative blood loss, total number of lymph nodes cleared, total number of metastatic lymph nodes cleared, and postoperative hospital stay.

In the Meta-analysis on the application of ICG NIR light imaging-guided lymph node dissection for minimally invasive radical gastric cancer surgery time, a total of 17 papers were included, and the heterogeneity test I²=95.7%>50% and P=0.0001<0.1 for Q-test suggested a high heterogeneity among the papers selected for this study, and the results of sensitivity analysis showed that none of the papers would have a strong For failure to analyze the source of heterogeneity, the random effects model was chosen to merge the effect sizes. The results were as follows: the MD of the effect size after Meta-combination was -5.799 (-16.251 to 4.653), but there was no significant difference in time to surgery in the ICG group compared to the non-ICG group (Z=1.09, p=0.277 > 0.05). In the Meta-analysis on intraoperative blood loss, a total of 11 papers were included, and after the heterogeneity test I²=95.2% > 50% and p=0.0001 < 0.1 for Q-test, suggesting a high heterogeneity between the papers selected for this study, and the results of sensitivity analysis showed that none of the papers would have a strong effect on the study results, and because the source of heterogeneity could not be analyzed, the choice of Meta-analysis was performed with a random effects model. The results were as follows: the effect size MD after Meta-combination was -14.554 (-25.424 to -3.683), and the effect size was significant (Z=2.62, p=0.009 < 0.01), suggesting a statistically significant 14.55 mL lower intraoperative blood loss in the ICG group compared with the non-ICG group. Regarding the total number of intraoperative lymph nodes cleared, 13 papers were included, and after the heterogeneity test I² = 79.1% > 50% and P < 0.1 for the Q test, suggesting a high heterogeneity among the papers selected for this study, and because the source of heterogeneity could not be analyzed, a random-effects model was selected for Meta-analysis. The results of the random-effects Meta-analysis were as follows: the effect size MD after Meta-combination was 5.575 (3.677-7.473), and the effect size was significant (Z=5.76, p<0.00001), indicating that the total number of intraoperative lymph node dissection was greater in the ICG group than in the non-ICG group, which was statistically significant. In the Meta-analysis of the number of intraoperative cleared metastatic lymph nodes, a total of seven papers were included, and after the heterogeneity test I² = 41.7% < 50% and p = 0.113 > 0.1 for the Q-test, suggesting that the effect of heterogeneity among studies can be ignored between the papers selected for this study, and the fixed-effect model was selected for Meta-analysis, and the results showed that the effect size after Meta-combination was 0.261 (-0.463 to 0.985), and the effect size (Z=0.71, p=0.480) was not statistically significant. In terms of postoperative length of stay, a total of 12 papers were included, and after heterogeneity test I²=62.1% >50% and p=0.002 <0.1 for Q-test, suggesting moderate heterogeneity among the literature selected for this study, and since the source of heterogeneity could not be analyzed, a random-effects model was selected for Meta-analysis. The results were as follows: the effect size MD after Meta-combination was -0.665 (-1.108 to -0.222) and the effect size was significant (Z=2.94, p<0.05), and the postoperative hospital stay of patients in the ICG group after treatment was significantly 0.665 d lower than in the non-ICG group, i.e., the intervention effect was significant (**Figures 4**, **5**).

**Figure 6:**
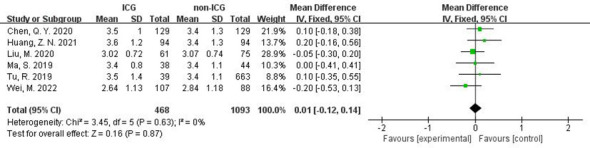
Forest plot assessment of recovery of first postoperative gas in the ICGFL group versus the non-ICGFL group.

**Figure 7:**
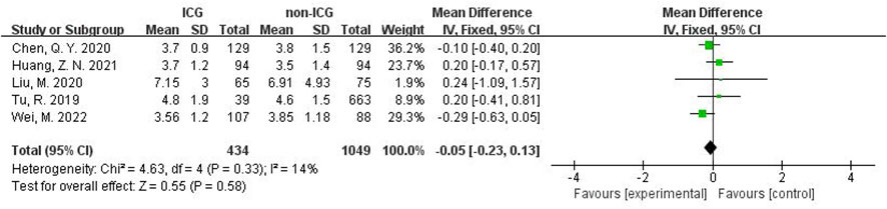
Forest plot assessment of time to first postoperative fluid intake in the ICG and non-ICG groups.

In addition, in the Meta-analysis on the application of ICG NIR light imaging-guided lymph node dissection for the recovery of the first gastric vent after minimally invasive radical gastric cancer surgery and the time to the first postoperative fluid intake, six and five papers were included, respectively, and the results of the heterogeneity test were I²=0%<50% and P=0.63>0.1 for Q-test and I²=14%<50% and P=0.33>0.1 for Q-test, respectively. It is suggested that there is no heterogeneity between the literature selected for both studies, so the fixed-effects model was selected for Meta-analysis. The results of the fixed-effects Meta-analysis were as follows: MD 0.01 (-0.12~-0.14), P=0.87>0.05, MD -0.05 (-0.23~0.13), P=0.58>0.05, respectively, suggesting that the time to first postoperative gas and time to first postoperative fluid intake were not significantly changed in the ICG group after treatment compared with the non-ICG group. Due to the small amount of included literature, a larger sample size of evidence-based medical evidence is needed to confirm this (**Figures 6**, **7**).

### 3.4 Subgroup analysis

Based on consideration of significant heterogeneity in effect sizes reflecting surgical and postoperative recovery, including operative time, intraoperative blood loss, total number of lymph nodes cleared, total number of metastatic lymph nodes cleared, and postoperative hospital stay, possibly due to differences in baseline parameters between the ICG and control groups (e.g., BMI, extent of gastrectomy, extent of lymph node clearance, tumor size, and TNM stage), as well as between-study differences, including study design, country, sample size, surgical approach, and duration of ICG use. This meta performed the corresponding subgroup analysis on both surgical approach and extent of gastrectomy.

**Figure 8:**
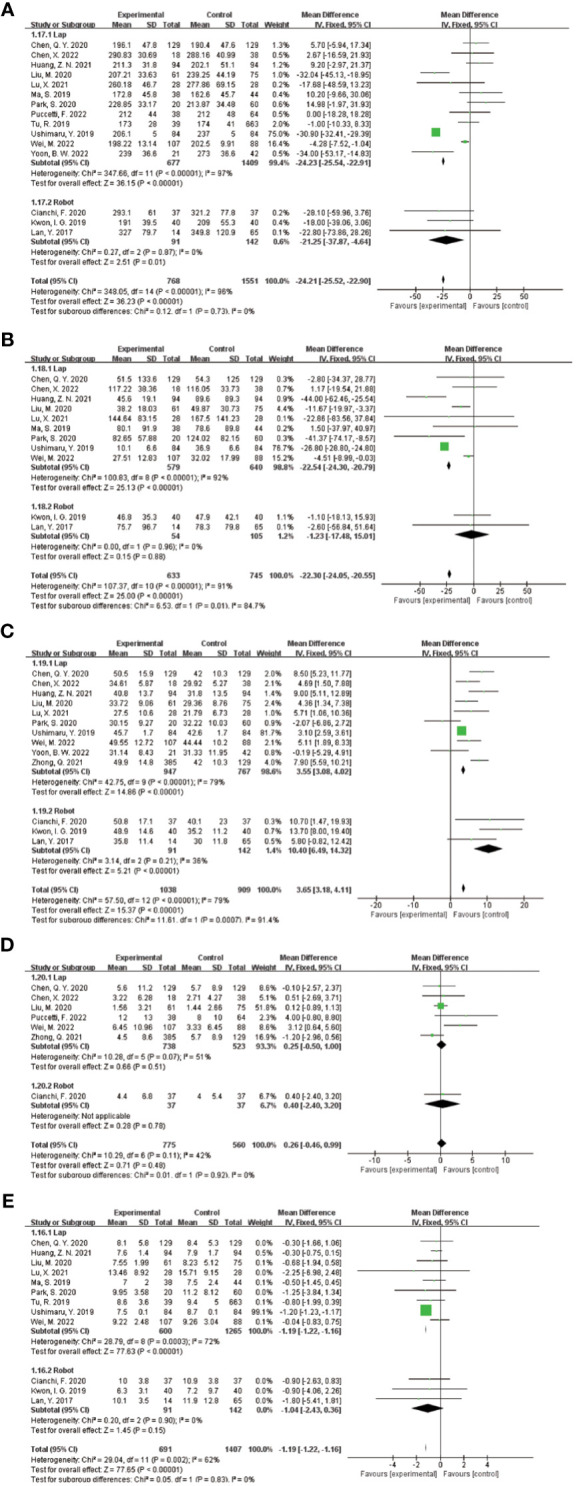
Results of subgroup analysis based on the correlation between laparoscopic and robotic minimally invasive surgical approaches. **(A–E)** in order of operative time, intraoperative blood loss, total number of lymph nodes cleared, number of metastatic lymph nodes cleared, and postoperative hospital stay.

In the stratified analysis of surgical approach (**Figure 8**), the use of ICG reduced the operative time in the Robot group by 21.25 min (95% CI -37.87 to -4.64), with a statistically significant p=0.01 <0.05; the total number of LNs recovered increased by 10.40 in the Robot group (95% CI 6.49~14.32), P<0.00001, suggesting a statistically significant increase.

**Figure 9:**
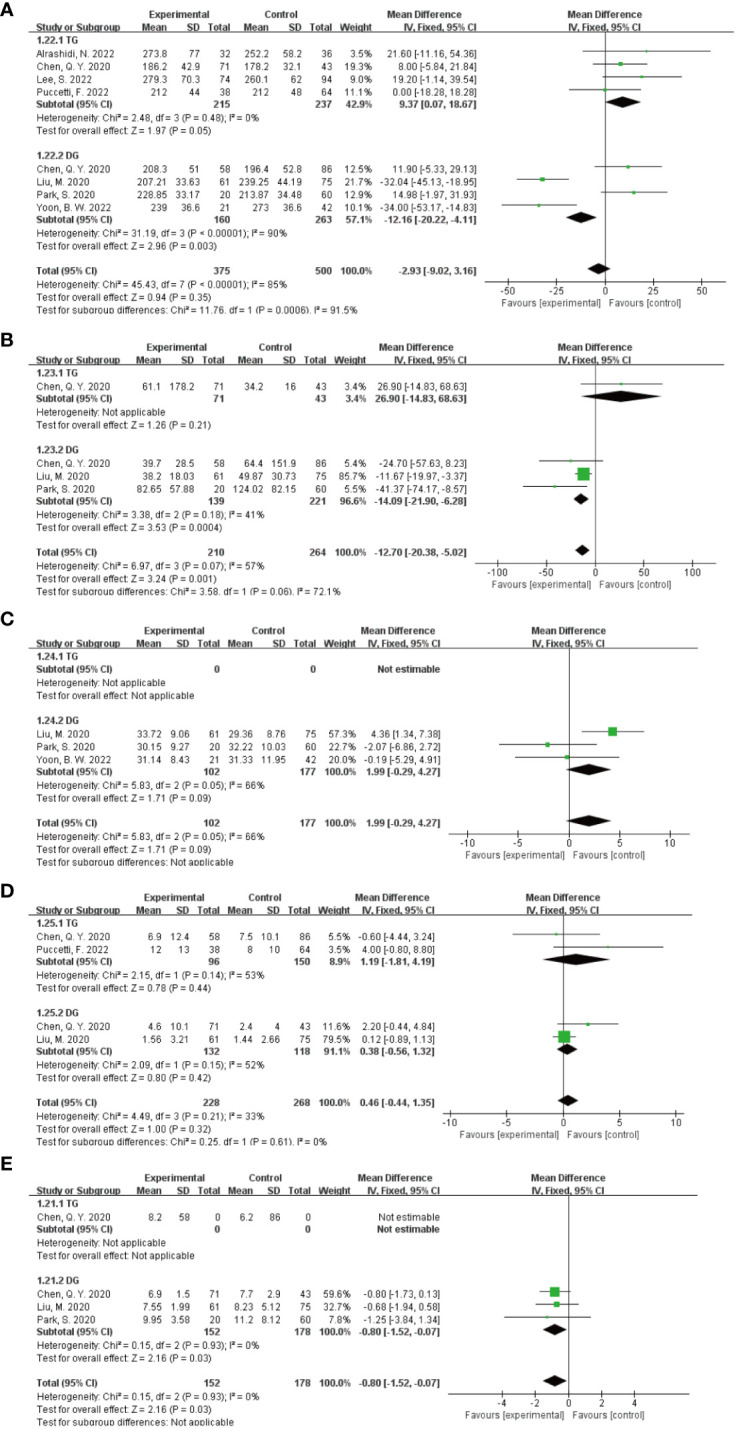
Results of subgroup analysis based on the extent of gastrectomy. **(A–E)** in order of operative time, intraoperative blood loss, total number of lymph nodes dissected, number of metastatic lymph nodes dissected, and postoperative hospital stay.

In the subgroup analysis of the extent of gastrectomy, divided into DG and TG groups, the results of the forest plot (**Figure 9**) showed that in the DG group, the application of ICG NIR light imaging-guided lymph node dissection reduced the postoperative hospital stay by 0.8d (95% CI -1.52~-0.07),P=0.03, which was statistically significant; the application of ICG NIR light imaging-guided lymph node dissection reduced reduced intraoperative blood loss by 14.09 mL (95% CI -20.90~-6.28),P=0.0004; in the TG group, the operative time was reduced by 9.37 min (95% CI 0.07~18.67),P=0.05, which was statistically significant. In addition, better results were obtained in each study by sensitivity analysis.

### 3.5 Publication bias test

Funnel plot, Egger’s method and Trim’s method were used to assess potential publication bias in the primary outcome summary analysis.

#### 3.5.1 Short-term postoperative prognosis

**Figure 10:**
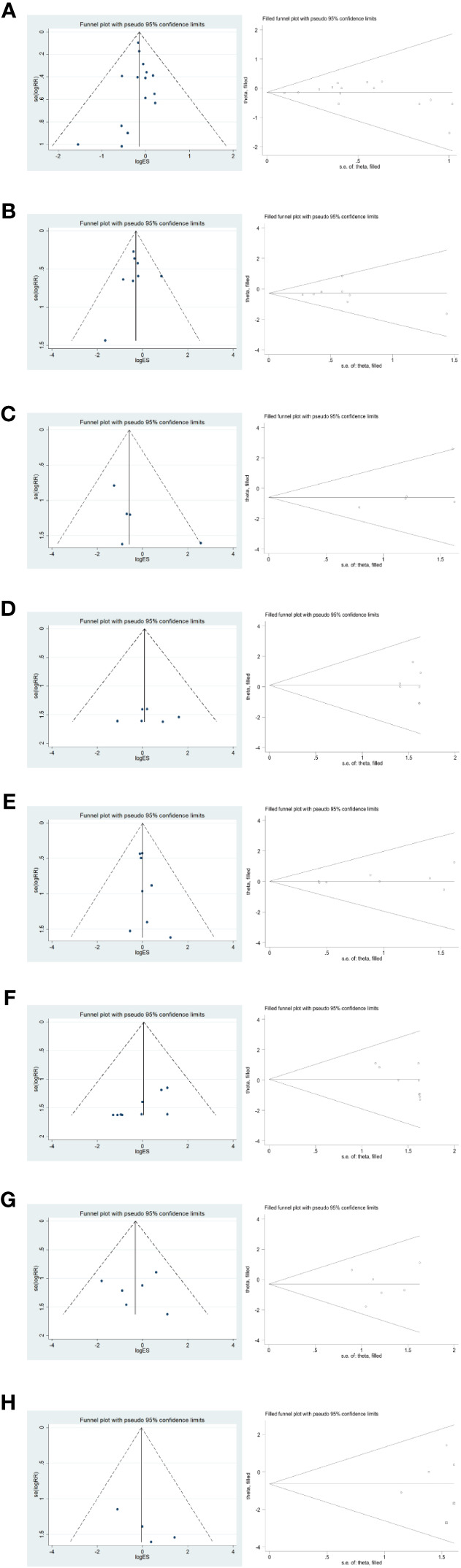
Funnel plot and Trim method to assess each outcome effect size for short-term postoperative prognosis. **(A–H)** in order of total complications, Clavien-Dindo grade II or higher complication rate, postoperative abdominal infection, abdominal bleeding, pneumonia, anastomotic fistula, postoperative gastric emptying disorder, and postoperative complication of intestinal obstruction.

To verify the publication bias for each outcome effect size (total complications and incidence of Clavien-Dindo grade II or higher complications, postoperative abdominal infection, abdominal bleeding, pneumonia, anastomotic fistula, postoperative gastric emptying disorder, and postoperative complications of intestinal obstruction) for short-term postoperative prognosis, the following funnel plot was plotted (**Figure 10**); furthermore, the funnel plot was subjected to The Egger method yielded p-values of 0.778, 0.864, 0.210, 0.710, 0.239, 0.018, 0.982, and 0.105, respectively, all of which were greater than 0.05. The stability of the combined results was also assessed by the Trim method, which is a cut-and-patch method. The results showed that there was a small bias in the pooled analysis of each outcome effect of short-term postoperative prognosis, with good reliability.

#### 3.5.2 Surgical and postoperative recovery

**Figure 11:**
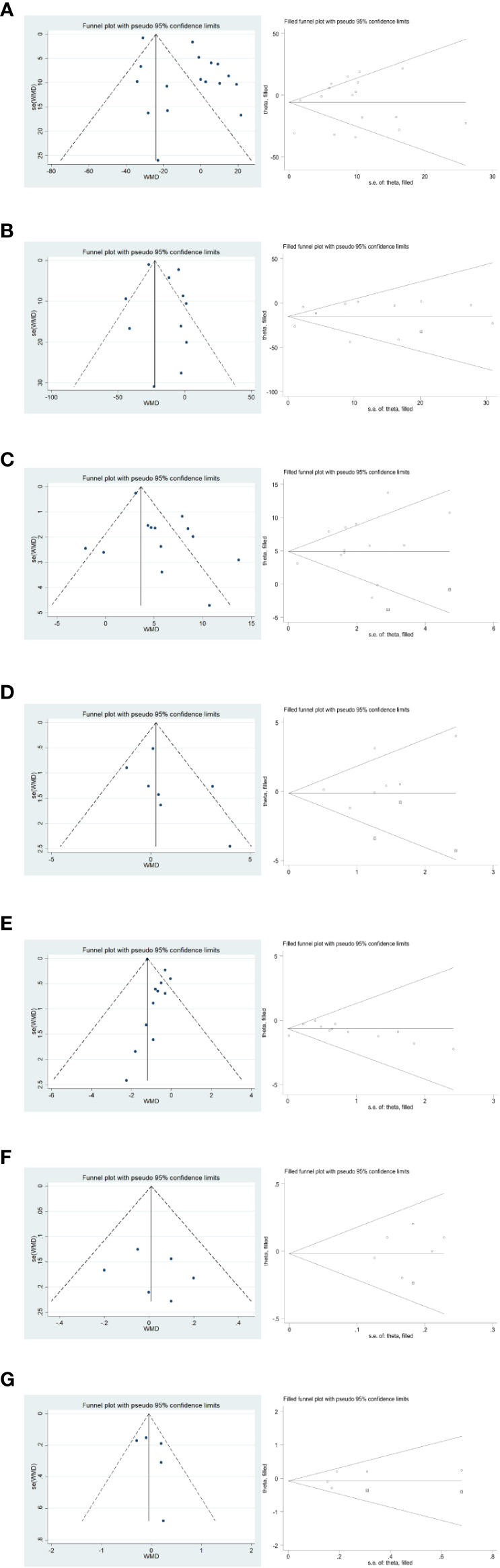
Funnel plot and Trim method to assess the outcome effect sizes for surgery and postoperative recovery. **(A–G)** in order of surgery time, intraoperative blood loss, total number of lymph nodes cleared, total number of metastatic lymph nodes cleared, postoperative hospitalization time, postoperative time to first gas, and postoperative time to first fluid intake.

In the publication bias validation of the outcome effect measures for surgery and postoperative recovery (**Figure 11**), the Egger method for the four effect measures of blood loss from surgery, number of metastatic lymph nodes cleared, time to first postoperative gas, and time to first fluid intake yielded P values of 0.265, 0.280, 0.609, and 0.434 > 0.05, respectively, with no significant publication bias; time to surgery The p-values of 0.020, 0.046, and 0.047 < 0.05 for postoperative hospital stay and total number of lymph nodes cleared by Egger’s method, respectively, suggested that there was some publication bias for the combined results. The stability of the combined results was also assessed by the Trim method, which is a cut-and-patch method; among them, the results did not change statistically after the inclusion of two dummy data in the total number of cleared lymph nodes, so the combined results were reliable and stable.

## 4 Discussion

The current meta-analysis demonstrates that the utilization of ICG NIR technology for guiding lymph node dissection in minimally invasive radical gastric cancer leads to improvements in short-term postoperative complications, particularly in reducing the incidence of complications classified as Clavien-Dindo grade II or higher. Moreover, the application of ICG technology in minimally invasive radical gastric cancer enhances the intraoperative clearance of tumor-invaded lymph nodes. Furthermore, ICG NIR light imaging-guided lymph node dissection proves beneficial in minimizing postoperative metastasis and recurrence, with particular advantages observed in robot-assisted radical gastric cancer surgery. Additionally, there is a potential reduction in operative time with the adoption of ICG-guided lymph node dissection. Overall, the findings suggest that incorporating ICG NIR light imaging-guided lymph node dissection in minimally invasive radical gastric cancer surgery not only improves short-term postoperative outcomes but also optimizes surgical procedures, such as decreasing operative time and reducing estimated blood loss. However, further investigation is warranted to assess the long-term survival advantages.

The application of NIR light imaging technology to guide lymph node dissection is contingent upon proficient and standardized minimally invasive radical gastric cancer surgery, with indications and contraindications determined based on laparoscopic or robotic surgical protocols, taking into account the patient’s history of ICG allergy. This underscores the vast potential of ICG in the realm of minimally invasive surgery. Previous studies have indicated that a higher number of dissected lymph nodes within a defined clearance range correlates with improved 5-year recurrence-free survival (RFS) and overall survival (OS) in patients with gastric cancer (36–38). Notably, both the Union for International Cancer Control (UICC) and the National Comprehensive Cancer Network (NCCN) guidelines stipulate that patients with radical gastric cancer should have a minimum of 15 lymph nodes dissected to achieve accurate staging (39). In the context of minimally invasive surgery for advanced gastric cancer, achieving thorough and effective perigastric lymph node dissection poses challenges due to the intricate anatomy and abundant blood supply of the stomach, particularly in patients with a high BMI. Conventional naked-eye lymph node dissection often results in a high rate of lymph node noncompliance (15). Consequently, enhancing the number of dissected lymph nodes and the detection of positive lymph nodes within the designated clearance area, and achieving accurate staging, subsequent treatment options, and improved prognosis for patients, have become focal points for surgical consideration.

Among the 19 studies included in this meta-analysis, D2 lymph node dissection, which represents the minimum standard for lymph node dissection extent, has consistently demonstrated improved survival outcomes in cancer patients (40). However, ensuring quality control of intraoperative lymph node dissection and rectifying deviations in lymph node staging are crucial elements for accurate cancer staging (41). In this regard, the application of lymph node tracer techniques has emerged as a valuable approach (42). In recent years, various lymph node tracers have been increasingly reported in minimally invasive procedures, including methylene blue (43), carbon nanoparticle suspension injection (CNSI) (44) and indocyanine green (ICG) (45). When compared to other lymph node tracers, ICG stands out for several reasons. It is relatively non-toxic and safe, offering an advantage in terms of patient well-being. Furthermore, it is cost-effective, with a price tag that is less than one-tenth of carbon nanoparticles (46, 47). Importantly, unlike other lymph node tracers, ICG exhibits minimal leakage from the injection site, resulting in less interference with the surgical field and facilitating smoother procedures (48). ICG NIR technology, with its specific fluorescence wavelength characteristics, exhibits a notable background contrast effect and deeper penetration depth (47). This enables real-time visualization of lymph nodes during intraoperative procedures and facilitates precise lymph node clearance in function-preserving surgeries. While the advantages of ICG NIR technology in terms of the total number of cleared lymph nodes are well established, its impact on minimally invasive surgery time, intraoperative bleeding, and short-term postoperative complications remains uncertain. The findings of this meta-analysis revealed that the addition of ICG NIR technology did not result in an increase in operative time, intraoperative blood loss, or postoperative hospital stay. On the contrary, improvements were observed in these parameters. Furthermore, although there was no statistically significant difference in the overall complication rate, postoperative abdominal infection, abdominal bleeding, pneumonia, anastomotic fistula, postoperative gastric emptying disorder, and postoperative complications of intestinal obstruction, the incidence of postoperative complications classified as Clavien-Dindo grade II or higher was significantly reduced in the ICG NIR technology group (P=0.05). This reduction has the potential to significantly shorten patients’ postoperative recovery time and enhance their postoperative quality of life. It is important to acknowledge that the study results did not demonstrate a statistically significant difference in the number of cleared metastatic lymph nodes between the ICG and non-ICG groups. There are several possible reasons for this observation:①The lymph nodes visualized through ICG fluorescence only indicate lymph nodes receiving lymphatic fluid return from the surrounding tumor tissue. They do not provide specific visualization of metastatic lymph nodes. As a result, the surgeon is faced with an independent decision-making process regarding the clearance of potential metastatic lymph nodes (49); ②Based on the surgeon’s experience, it may be deemed more effective to completely clear metastatic lymph nodes under conventional naked-eye conditions.These factors contribute to the absence of a significant difference in the number of metastatic lymph nodes cleared between the ICG and non-ICG groups in the study.

To assess the heterogeneity among the studies and examine the consistency of the combined results, this meta-analysis conducted subgroup analyses based on the surgical approach and the extent of gastrectomy. The findings of the subgroup analysis are as follows:In the Robot group, the use of ICG NIR technology resulted in a shorter operative time and an increased total number of recovered lymph nodes;For patients undergoing minimally invasive distal gastric cancer radical resection, the addition of ICG NIR technology led to statistically significant reductions in operative time, intraoperative blood loss, and postoperative hospital stay (P < 0.01).In conclusion, the application of ICG NIR technology in robotic-assisted radical gastric cancer surgery and minimally invasive distal gastric cancer radical surgery can provide more significant advantages.

This meta-analysis has several limitations that should be acknowledged. Firstly, although two randomized controlled trials were included, most of the other studies were retrospective cohort studies, which increases the risk of selective bias. Secondly, there was high heterogeneity in the outcome effect sizes of the continuous variables among the included studies. Subgroup analysis and sensitivity analysis were performed to explore the source of heterogeneity, and a random-effects model was used when the cause of heterogeneity could not be identified, which improved the credibility of the data. Thirdly, the influence of high BMI on the difficulty of lymph node dissection in minimally invasive radical gastric cancer surgery was not analyzed separately due to insufficient data. Fourthly, the majority of the literature included in this meta-analysis focused on preoperative submucosal injection of ICG, while the number of cases with intraoperative submucosal injection was limited, leading to potential bias in the data.

## 5 Conclusion

In conclusion, this meta-analysis provides strong evidence supporting the use of ICG NIR imaging-guided lymph node dissection in minimally invasive radical gastric cancer surgery. The findings indicate that this technique is safe, feasible, and effective in reducing the incidence of postoperative complications above Clavien-Dindo grade II. It also improves the quality control of lymph node dissection and corrects staging deviations. The combination of ICG NIR imaging with robotic surgical systems shows even greater potential for improving short-term patient outcomes while maintaining surgical safety.However, it is important to note that the impact of this technology on long-term patient survival still needs to be further investigated. Future studies should focus on conducting multicenter, high-quality randomized controlled trials to validate the benefits of ICG NIR imaging in improving long-term survival outcomes for patients undergoing minimally invasive radical gastric cancer treatment.

## Author contributions

SN: Conceptualization, Writing – original draft, Methodology, Resources, Software, Visualization. YL: Conceptualization, Writing – original draft, Methodology, Resources, Software, Visualization. DL: Data curation, Formal Analysis, Writing – original draft. YS: Data curation, Formal Analysis, Writing – original draft. YZ: Data curation, Formal Analysis, Writing – original draft, Funding acquisition. ZL: Data curation, Formal Analysis, Writing – original draft. SZ: Writing – review & editing, Validation. TW: Writing – review & editing, Funding acquisition, Validation.

## Supplementary material

Supplementary Table 1 The search strategy for each database.

Supplementary Table 2 The original dataset used in the study.
