# Supplementary material for: Effect of indocyanine green near-infrared light imaging technique guided lymph node dissection on short-term clinical efficacy of minimally invasive radical gastric cancer surgery: a meta-analysis
Source: Front Oncol. 2023 Sep 11;13:1257585. doi: 10.3389/fonc.2023.1257585 (PMC10520705; doi:10.3389/fonc.2023.1257585)
Supplement: Supplementary Table 1 — The search strategy for each database. [file Table_1.docx]

**Table S1** The search strategy for each database.

| **Database** | **Search strategy** |
| --- | --- |
| PubMed | **(((((((((((((((((((Neoplasm, Stomach[Title/Abstract]) OR (Stomach Neoplasm[Title/Abstract])) OR (Neoplasms, Stomach[Title/Abstract])) OR (Gastric Neoplasms[Title/Abstract])) OR (Gastric Neoplasm[Title/Abstract])) OR (Neoplasm, Gastric[Title/Abstract])) OR (Neoplasms, Gastric[Title/Abstract])) OR (Cancer of Stomach[Title/Abstract])) OR (Stomach Cancers[Title/Abstract])) OR (Gastric Cancer[Title/Abstract])) OR (Cancer, Gastric[Title/Abstract])) OR (Cancers, Gastric[Title/Abstract])) OR (Gastric Cancers[Title/Abstract])) OR (Stomach Cancer[Title/Abstract])) OR (Cancer, Stomach[Title/Abstract])) OR (Cancers, Stomach[Title/Abstract])) OR (Cancer of the Stomach[Title/Abstract])) OR (Gastric Cancer, Familial Diffuse[Title/Abstract])) OR ("Stomach Neoplasms"[Mesh])) AND (("Indocyanine Green"[Mesh]) OR (((((((Green, Indocyanine[Title/Abstract]) OR (Wofaverdin[Title/Abstract])) OR (Vophaverdin[Title/Abstract])) OR (Vofaverdin[Title/Abstract])) OR (Cardio-Green[Title/Abstract])) OR (Cardio Green[Title/Abstract])) OR (Cardiogreen[Title/Abstract])))** |
| Web of Science | **(TS=(Indocyanine Green OR Green, Indocyanine OR Wofaverdin OR Vophaverdin OR Ujoveridin OR Vofaverdin OR Cardio-Green OR Cardio Green OR Cardiogreen)) AND (TS=(Stomach Neoplasms OR Neoplasm, Stomach OR Stomach Neoplasm OR Neoplasms, Stomach OR Gastric Neoplasms OR Gastric Neoplasm OR Neoplasm, Gastric OR Neoplasms, Gastric OR Cancer of Stomach OR Stomach Cancers OR Gastric Cancer OR Cancer, Gastric OR Cancers, Gastric OR Gastric Cancers OR Stomach Cancer OR Cancer, Stomach OR Cancers, Stomach OR Cancer of the Stomach OR Gastric Cancer, Familial Diffuse))** |
| Cochrane Library | **#1. (Indocyanine Green):ab,ti,kw OR (Green, Indocyanine):ab,ti,kw OR (Wofaverdin):ab,ti,kw OR (Vophaverdin):ab,ti,kw OR (Ujoveridin):ab,ti,kw OR (Vofaverdin):ab,ti,kw OR (Cardio-Green):ab,ti,kw OR (Cardio Green):ab,ti,kw OR (Cardiogreen):ab,ti,kw** |
|  | **#2. (Stomach Neoplasms):ab,ti,kw OR (Neoplasm, Stomach):ab,ti,kw OR (Stomach Neoplasm):ab,ti,kw OR (Neoplasms, Stomach):ab,ti,kw OR (Gastric Neoplasms):ab,ti,kw OR (Gastric Neoplasm):ab,ti,kw OR (Neoplasm, Gastric):ab,ti,kw OR (Neoplasms, Gastric):ab,ti,kw OR (Cancer of Stomach):ab,ti,kw OR (Stomach Cancers):ab,ti,kw OR (Gastric Cancer):ab,ti,kw OR (Cancer, Gastric):ab,ti,kw OR (Cancers, Gastric):ab,ti,kw OR (Gastric Cancers):ab,ti,kw OR (Stomach Cancer):ab,ti,kw OR (Cancer, Stomach):ab,ti,kw OR (Cancers, Stomach):ab,ti,kw OR (Cancer of the Stomach):ab,ti,kw OR (Gastric Cancer, Familial Diffuse):ab,ti,kw** |
|  | **#3. #1 AND #2** |
| Embase | **#1. 'Green, Indocyanine':ab,ti OR 'Wofaverdin':ab,ti OR 'Vophaverdin':ab,ti OR 'Ujoveridin':ab,ti OR 'Vofaverdin':ab,ti OR 'Cardio-Green':ab,ti OR 'Cardio Green':ab,ti OR 'Cardiogreen':ab,ti** |
|  | **#2. 'Neoplasm, Stomach':ab,ti OR 'Stomach Neoplasm':ab,ti OR 'Neoplasms, Stomach':ab,ti OR 'Gastric Neoplasms':ab,ti OR 'Gastric Neoplasm':ab,ti OR 'Neoplasm, Gastric':ab,ti OR 'Neoplasms, Gastric':ab,ti OR 'Cancer of Stomach':ab,ti OR 'Stomach Cancers':ab,ti OR 'Gastric Cancer':ab,ti OR 'Cancer, Gastric':ab,ti OR 'Cancers, Gastric':ab,ti OR 'Gastric Cancers':ab,ti OR 'Stomach Cancer':ab,ti OR 'Cancer, Stomach':ab,ti OR 'Cancers, Stomach':ab,ti OR 'Cancer of the Stomach':ab,ti OR 'Gastric Cancer, Familial Diffuse':ab,ti** |
|  | **#3. #1 AND #2** |
